# Acute Respiratory Failure Is the Initial Manifestation in the Adult-Onset A3243G tRNALeu mtDNA Mutation: A Case Report and the Literature Review

**DOI:** 10.3389/fneur.2019.00780

**Published:** 2019-07-18

**Authors:** Xiaoli Pan, Lijun Wang, Guoqiang Fei, Jihong Dong, Chunjiu Zhong, Jiahong Lu, Lirong Jin

**Affiliations:** ^1^Department of Neurology, Zhongshan Hospital & Shanghai Medical College, Fudan University, Shanghai, China; ^2^Department of Neurology & Co-innovation Center of Neurodegeneration, Ruijin Hospital Affiliated to Shanghai Jiao Tong University School of Medicine, Shanghai, China; ^3^Department of Neurology, Huashan Hospital, Fudan University, Shanghai, China

**Keywords:** mitochondrial myopathy, A3243G mutation, respiratory failure, mtDNA, sedative drug

## Abstract

Isolated mitochondrial myopathy refers to the condition of mitochondrial disorders that primarily affect the skeletal muscle system. Here we report on a case of a patient who presented with acute respiratory failure as the initial and predominant clinical manifestation after using anesthetic drugs. The diagnosis of mitochondrial myopathy was made by histochemical findings of ragged red fibers with a modified Gomori trichrome Stain in the skeletal muscle biopsy and the genetic detection of an A3243G point mutation in the tRNA^Leu^ (UUR) gene of mitochondrial DNA (mtDNA) in a peripheral blood specimen. The patient revealed a benign clinical outcome with ventilator assistance and a cocktail treatment. Further, we performed a literature review on patients with respiratory failure as the early and predominant manifestation in adult-onset isolated mitochondrial myopathy. Eleven cases in nine studies (including our case) have been reported, and five of whom underwent DNA analysis all harbored the A3243G mutation in the tRNA^Leu^ gene of the mtDNA. Use of sedative drugs tends to induce acute respiratory failure in such cases.

## Introduction

Mitochondria are the cellular organelles responsible for energy production, especially by completing oxidative phosphorylation. Organ systems that rely most on aerobic metabolism, such as the brain, heart and the skeletal muscle, are more inclined to be influenced by mitochondrial dysfunction. Mitochondrial disease affects multiple organs with various severity, ranging from skeletal muscles alone, or the central nervous system to multiorgan system impairment with myopathy, and presents with heterogeneous clinical manifestations (1). The term of isolated mitochondrial myopathy refers to the condition where mitochondrial disorders primarily affect the skeletal muscle system. The clinical features of isolated mitochondrial myopathy can be highly variable, including mild exercise intolerance, fatigue, muscle weakness, myalgia, mild elevated serum creatine kinase and more rare, rhabdomyolysis (2).

Respiratory failure in mitochondrial myopathy usually occurs at the late stage of the disease, and is associated with deterioration of respiratory muscle weakness (3, 4). However, an acute episode of respiratory failure as the initial and predominant clinical feature in adult-onset mitochondrial myopathy is rarely reported and is easily misdiagnosed as other neuromuscular disorders, such as Guillain-Barre syndrome (GBS) and myasthenia gravis (MG). Here we report on a 52-years-old man who presented with acute respiratory failure as the initial and predominant manifestation after receiving a painless gastroscopy and colonoscopy examination with use of anesthetic drugs. He was diagnosed with mitochondrial myopathy by histochemical findings of ragged red fibers (RRF) in the skeletal muscle biopsy and an A3243G point mutation of the mitochondrial tRNA^Leu^ gene in a peripheral blood specimen. Further, we performed a literature review on clinical features of adult-onset isolated mitochondrial myopathy with abrupt episodes of respiratory dysfunction as the early and predominant manifestation. Eleven cases have been reported in nine studies (including our study), and five of whom received DNA analysis all carried the A3243G mutation in the tRNA^Leu^ gene of the mtDNA (3, 5–11).

## Case Report

A 52-years-old man was transferred to our intensive care unit in July 2016 because of an acute episode of respiratory failure after using anesthetic drugs 8 days earlier. He felt easily fatigued and developed insidiously limb weakness in the previous 6 months. He attributed this to toothache resulting in a poor appetite. However, he was still able to maintain his daily life and keep on working. His family noticed that he lost 7.5 kg of body weight in 6 months and took him to the gastroenterology department of a local hospital. He received a painless gastroscopy and colonoscopy examination using propofol on 19 July 2016. He regained consciousness very slowly for over forty min and was found to have hypoxemia with a blood oxygen saturation degree (SO_2_) of 86%. After expectant treatment of oxygen inhalation, he recovered to a relative stable condition and drove home. He developed increasing shortness of breath in the following days, which eventually deteriorated into acute respiratory failure. Arterial blood gas (ABG) analysis showed a pH of 7.25, and a PaCO_2_ of 86.25 mmHg. Although the PaO2 level was in a normal range by treatment of oxygen inhalation, the diagnosis of type II respiratory failure was considered because of hypercapnia. A pulmonary function test suggested a moderate restrictive ventilator impairment. No evidence of pneumonia or bronchitis was found through Computed Tomography (CT) scanning. Since he developed recurrent episodes of hypoxemia, hypercapnia and delirium, he received artificial ventilatory assistance on July 26th. Next day, he was transferred to our hospital.

He denied a family history of neuromuscular disease, central nervous system dysfunction, genetic disorders, diabetes mellitus, or vision and hearing dysfunction. His mother was emaciated and suddenly passed away of unknown reason at the age of 60. His siblings were reportedly healthy.

On admission the patient was in a mild agitated state. He looked very thin and his height was 1.65 meters and weighed 47 Kg. His body temperature was 36.8°C, pulse rate 90/min, respiratory rate 25/min, and blood pressure 116/69 mmHg. The SO_2_ level was 100% with oxygen inhalation by nasal mask at 3.0 L/min. No abnormality was found in the respiratory and cardiovascular systems. A neurological examination showed that cranial nerves function was normal. There was no ptosis, external ophthalmoplegia, diplopia or facial weakness. The proximal upper and lower limb muscle power was mildly decreased (Medical Research Council grading criterion, grade IV) and his distal muscle strength was approximately normal. His tendon reflexes were diminished. No abnormalities in the long tract, sensitive system, meningeal irritation or cerebellar signs were noted.

Laboratory investigations revealed normal levels of liver function, kidney function, blood glucose, blood ammonia, serum electrolytes, blood clotting function, serum creatine phosphokinase, thyroid function, rheumatologic antibodies, tumor biomarkers, folate, and vitamin B12. The peripheral white blood cell count was normal, but the percent of segmented neutrophils increased to 84.6%. ABG analysis showed a pH of 7.24, a PaCO_2_ of 120 mmHg and a PaO_2_ of 198 mmHg with O_2_ inhalation. Blood lactate level at rest in the first test of our hospital was normal but fluctuated from normal to >12 mmol/L (0.7–2.1 mmol/L) in the successive tests. Because of a presumptive diagnosis of myasthenic crisis, he received pyridostigmine for a week without any improvement. No abnormalities were found in the levels of the serum anti-acetylcholine receptor (AChR) antibodies.

Electrocardiogram, a computed tomography scan of the chest and Magnetic Resonance Imaging of the head and cervical spinal cord were normal (Figure S1). There was no abnormality in the repetitive nerve stimulation examination and the motor and sensory nerve conduction velocity. Needle electromyography (EMG) revealed small, short-duration and polyphasic motor unit action potentials in the musculus biceps brachii, deltoid, quadriceps femoris, sternocleidomastoid and rectus abdominis bilaterally, which is consistent with myopathic disorders. Tandem mass spectrometry analysis for organic acid in blood and urine was performed to detect lipid storage myopathy and the results remained in a normal range.

**Figure 1:**
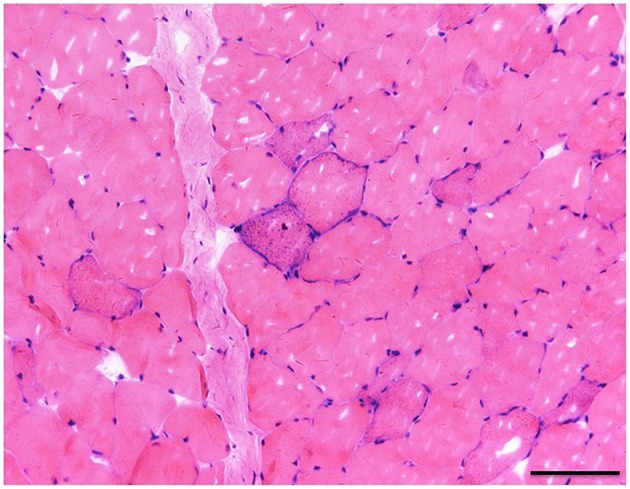
The fiber size was variable and fiber shapes were mildly irregular in Hematoxylin-eosin staining. There was one clear RRF (×20), bar = 100μ.

**Figure 2:**
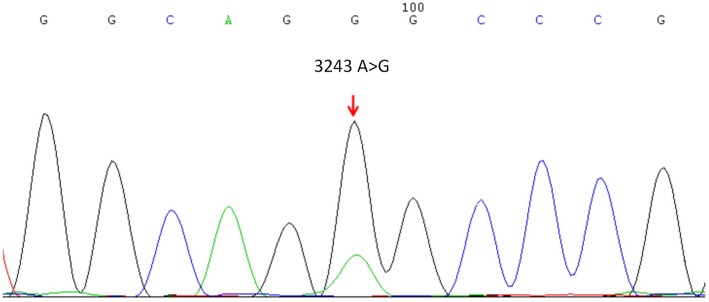
Mitochondrial DNA sequencing revealed the A3243G point mutation in the mitochondrial tRNA^Leucine^ gene.

A muscle biopsy of the left musculus biceps brachii was performed. Myopathic alterations were observed but no evidence of obvious inflammation, necrosis and degeneration was found. The fiber size was variable and fiber shapes were mildly irregular by Hematoxylin-eosin staining (Figure 1). Some RRF were evident as shown in Figure 1. Increased enzyme activities in some muscle fibers were observed using cytochrome c oxidase (COX). Expressions of MHC-I, R, C, N-dystrophin, α, β, γ-sarcoglycan, and dysferlin were normal. No abnormalities were seen in the staining of NADH, Periodic Acid Schiff (PAS), oil red O (ORO), and Adenosine Triphosphate (ATP) synthase. Screening for putative point mutation by polymerase chain reaction (PCR) revealed an A3243G mutation (88%) in the mitochondrial tRNA^Leu^ gene from total DNA extracted from the peripheral blood specimen (Figure 2).

According to the findings of EMG and muscle biopsy, the patient was diagnosed as mitochondrial myopathy with mtDNA A3243G point mutation. He was given a cocktail treatment of vitamin-C, B1, riboflavin, coenzyme Q10, cobamamide, and L-carnitine. Non-invasive ventilatory support with bilevel positive airway pressure (BiPAP) therapy via a basal mask was continued for 22 days, and then the artificial ventilator assistance was only used at night. The follow-up ABG analysis results were normal. His mental state, body weight and exercise tolerance improved gradually. He was discharged with the ventilator. The patient was reviewed for more than 2 years. Nocturnal BiPAP had been well-tolerated and ABG analysis was performed every 2 weeks. His limb power regained to the normal extent and his weight increased to 60 kg.

## Discussion

Here we report on a case of a 52-years-old man who presented with acute respiratory failure as the predominant clinical manifestation after using anesthetic drugs. He was able to maintain daily activity in spite of developing mild exercise intolerance and being easily fatigued before his admission. The patient was diagnosed with mitochondrial myopathy on the basis of the pathological findings of RRF in a muscle biopsy and genetic analysis of an A3243G point mutation in the tRNA^Leu^ (UUR) gene of mitochondrial DNA (mtDNA). He recovered gradually using ventilator assistance and a cocktail treatment and revealed a benign clinical outcome.

So far, adult-onset mitochondrial myopathy with acute episodes of respiratory failure as the early and predominant manifestation has rarely been reported. Only 11 cases including four males and seven females, in nine studies have been described (including our case, listed in Table 1) (3, 5–11). The onset age of the cases range widely from 16 to 70 years of age and all denied a history of mitochondrial myopathy. Although several cases reveled increased levels of serum creatine phosphokinase and lactate, the increased extent remains 2- or 3-fold of the normal level, which is different to other types of myopathy, such as inflammatory myopathies.

**Table 1:** Literature review on Respiratory failure as the predominant manifestation in adult-onset mitochondrial myopathy.

**Author**	**No**.	**Sex**	**Age**	**Family history**	**Onset of age**	**Lactate (mmol/L)**	**Creatine kinase (U/L)**	**Pulmonary function/arterial blood gas analysis**	**Electromyography**	**Muscle biopsy**	**Genetic or biochemical analysis**
Kim et al. (5)	2	Female	16	No	16	3.6 (0.5–2.2)	–	Both Type II respiratory failure	Myopathic alteration	Mitochondrial myopathy	–
		Male	22	No	19	–	Normal				–
Cros et al. (3)	2	Male	56	No	56	Normal	Case I: mild elevated (three times normal)	Case I: V/Q mismatching and alveolar hypoventilation;	Case I: Normal	Mitochondrial myopathy	Case I: Cytochrome oxidase deficiency;
		Female	70	No	70	Normal	Case II: Normal	Case II: restrictive ventilator impairment	Case II: Myopathic alteration		Case II: succinate-cytochrome c reductase defect
								Both Type II respiratory failure			
O'Brien et al. (6)	1	Female	27	No	27	Normal	Normal	Restrictive ventilatory defect/Type II respiratory failure	Normal	Mitochondrial myopathy	Cytochrome oxidase deficiency
Yang et al. (7)	1	Female	55	No	55	3.0	Normal	Mild restrictive ventilatory defect/Type II respiratory failure	Myopathic alteration	Mitochondrial myopathy	m.3243A>G
Chang et al. (8)	1	Male	32	No	30	2-fold of normal	2-fold of normal	Type II respiratory failure	Normal	Mitochondrial myopathy	m.3243A>G
Guo et al. (10)	1	Female	47	No	45	3.3 (0.4–1.7)	208 (18–198)	Restrictive ventilatory defect/Type II respiratory failure	Myopathic alteration	Mitochondrial myopathy	–
Amornvit et al. (9)	1	Female	20	No	20	9.1 (0.5–2.2)	Normal	Mild restrictive ventilatory defect	Myopathic alteration	Mitochondrial myopathy	m.3243A>G
Naddaf and Milone (11)	1	Female	42	Not mentioned	Not mentioned	Not mentioned	1.2 times upper limit of normal	Restrictive ventilatory defect	Myopathic alteration	Mitochondrial myopathy	m.3243A>G
The case reported here	1	Male	52	No	52	Normal	Normal	Moderate restrictive ventilatory defect	Myopathic alteration	Mitochondrial myopathy	m.3243A>G

All the cases presented with type II respiratory failure, the condition of hypercapnia and hypoxemia coexisting simultaneously, as the initial and predominant clinical manifestation on their admission. The patients may present with mild muscle weakness and exercise intolerance before, but these conditions were not the main complaints of their admission to the hospital. The pulmonary function test usually showed restrictive ventilatory defects. Most of the patients needed artificial ventilator assistance on admission. They were finally diagnosed with mitochondrial myopathy by muscular biopsy, biochemical and genetic analysis. RRF and abnormal mitochondria were mostly observed in the muscle biopsy and altered enzyme activities associated with the respiratory chain in mitochondria were found in the biochemical analysis in some cases.

We note that the same mutation, the m.3243A>G point mutation of mtDNA, was detected in five cases who received genetic determination. Population-based studies suggest the m.3243A>G mutation is the most common disease-causing mtDNA mutation, with a carrier rate of 1 in 400 people (12, 13). Studies have shown that mitochondrial disease remains the considerable variation in clinical features in patients with the m.3243A>G mutation. The m.3243A>G mutation is responsible for several classical syndromes, such as mitochondrial encephalopathy, lactic acidosis, and stroke-like episodes (MELAS), maternally inherited deafness and diabetes (MIDD), progressive external ophthalmoplegia (PEO) and Leigh syndrome. Other clinical phenotypes of mitochondrial disorders that cannot be classified as the above syndromes also detect this mutation, including isolated myopathy, cardiomyopathy, seizures, migraine, ataxia, cognitive impairment, bowel dysmotility and short stature (14). The A to G substitution leads to the pre termination of transcription and expression impeding of normal rRNA, thus compromising mitochondrial protein synthesis, ATP production and organic metabolism (15). Besides the A3243G mutation, the genetic defects of isolated myopathies can be caused by other abnormalities of mtDNA or nuclear DNA (nDNA) that affects the mitochondrial respiratory chain, such as m5698G>A, m618T>C, m5543T>C, 7526A>G in tRNA, thymidine kinase 2 (TK2) gene, or CoQ deficiency (16–21).

Exercise intolerance is the most common clinical feature in isolated mitochondrial myopathy. More often, respiratory failure occurs when the disease deteriorates to advanced periods (22). The sudden episodes of respiratory failure caused by isolated mitochondrial myopathies in the early stage is rarely described and easily misdiagnosed. Nine studies have reported 11 cases (including the case reported here) that were diagnosed with isolated mitochondrial myopathy with respiratory insufficiency as the primary and earliest clinical feature. Notably, the reduced hypoxic and hypercapnic ventilatory responses were out of proportion to their mild limb weakness. The specific mechanism of respiratory hypoventilation in mitochondrial myopathy is still unclear. Two possible pathophysiologic mechanisms may be involved in these patients: abnormality of the respiratory drive due to dysfunction of the respiratory centers in the brain stem (23, 24); weakness of the primary inspiratory muscles because of the mitochondrial insufficiency (5). Carroll et al. presumed that the depressed ventilatory drive of patients with mitochondrial myopathies was due to a CNS abnormality, possibly at the brainstem level (23). This was based on the lack of sufficient weakness to account for decreased ventilation, which was verified by Barohn et al.'s study (24). In addition, Kamakura et al. speculated that the increased abnormal mitochondria in the diaphragm may be involved in the recurrent episodes of respiratory failure (25).

On the basis of the unique clinical manifestation and the findings of the pulmonary tests of such patients, Amornvit has postulated that isolated mitochondrial myopathy predominantly affecting the respiratory muscles may be considered an uncommon clinical spectrum of the A3243G mitochondrial disease (9). Our study added new evidence to this rare clinical condition. Since acute episodes of respiratory failure caused by respiratory muscle weakness are life-threatening, isolated mitochondrial myopathy should be considered when no evidence of other neuromuscular disease exists, such as GBS, MG, Glycogen storage disease type II, and collagen VI disorders. Hence, isolated mitochondrial myopathy should be added to the differential diagnoses of neuromuscular respiratory failure.

Notably, the case in our study and another case reported in Yang's study (7) both presented with respiratory failure and a history of specific drug use, such as hypnotic and narcotic. The exact mechanism of how the sedative drugs induced the development of respiratory failure in insidious mitochondrial myopathy patients is unknown. Caroll and Barohn both observed that the depressed ventilatory drive of patients with mitochondrial myopathy was attributed to abnormalities in the respiratory control system located in the brain stem (23, 24). Since sedative drugs suppress the central nervous system, this may aggravate the reduced response to hypoxemia and hypercapnia. More attention should be paid on the prescription of sedative drugs in patients with isolated myopathy, who are inclined to respiratory failure.

## Conclusion

In conclusion, the case reported here presented with acute respiratory failure as the initial and predominant manifestation and the A3243G point mutation of mtDNA in isolated myopathy was detected. We expanded the evidence on a very uncommon condition, as a rare spectrum of isolated mitochondrial myopathy with the A3243G point mutation. Use of sedative drugs tends to induce respiratory failure in such cases.

## Data Availability

The datasets generated for this study are available on request to the corresponding author.

## Ethics Statement

The study was approved by the Committee on Medical Ethics of Zhongshan Hospital, Fudan University. Written informed consent was obtained from the patient for the publication of this case report. As this is a case report, without experimental intervention into routine care, no formal research ethics approval was required.

## Author Contributions

LJ and JL designed the case report. XP, LW, and GF performed the histochemical studies and wrote the article. JD performed the muscle biopsy. CZ revised the manuscript. All authors contributed to the manuscript revision and approved the final manuscript.

### Conflict of Interest Statement

The authors declare that the research was conducted in the absence of any commercial or financial relationships that could be construed as a potential conflict of interest.

## Supplementary Material

Figure S1 No abnormality was found in the head MRI scan.
