# Supplementary figures and images for: Acute Respiratory Failure Is the Initial Manifestation in the Adult-Onset A3243G tRNALeu mtDNA Mutation: A Case Report and the Literature Review
Source: Front Neurol. 2019 Jul 18;10:780. doi: 10.3389/fneur.2019.00780 (PMC6657224; doi:10.3389/fneur.2019.00780)

***Supplementary Material***

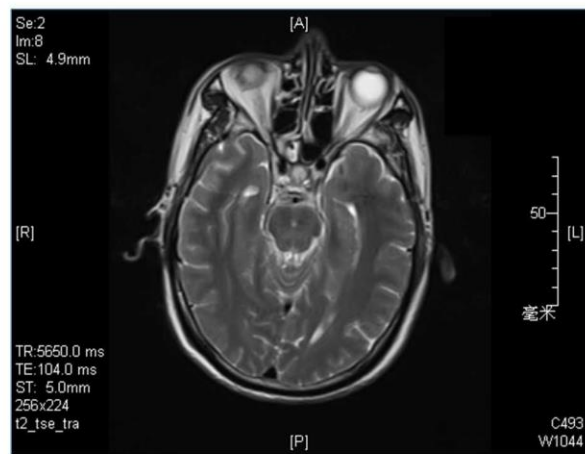

**Figure S1.** No abnormality was found in the head MRI scan.

Supplement: Figure S1 — No abnormality was found in the head MRI scan. [file Data_Sheet_3.pdf]
